# Vessel Delineation Using U-Net: A Sparse Labeled Deep Learning Approach for Semantic Segmentation of Histological Images

**DOI:** 10.3390/cancers15153773

**Published:** 2023-07-25

**Authors:** Lukas Glänzer, Husam E. Masalkhi, Anjali A. Roeth, Thomas Schmitz-Rode, Ioana Slabu

**Affiliations:** 1 Institute of Applied Medical Engineering, Helmholtz Institute, Medical Faculty, RWTH Aachen University, Pauwelsstraße 20, 52074 Aachen, Germany; glaenzer@ame.rwth-aachen.de (L.G.); husam.masalkhi@rwth-aachen.de (H.E.M.); smiro@ame.rwth-aachen.de (T.S.-R.); 2 Department of Visceral and Transplantation Surgery, University Hospital RWTH Aachen, Pauwelsstrasse 30, 52074 Aachen, Germany; aroeth@ukaachen.de; 3 Department of Surgery, Maastricht University, P. Debyelaan 25, 6229 Maastricht, The Netherlands

**Keywords:** deep learning, semantic segmentation, U-Net, histological images

## Abstract

**Simple Summary:**

In our study, we aimed to create an accurate segmentation algorithm of blood vessels within histologically stained tumor tissue using deep learning. Blood vessels are crucial for supplying nutrients to tumor cells, and accurately identifying them is essential for understanding tumor development and designing effective treatments. We conducted a comprehensive investigation by comparing various deep learning architectural methods. Additionally, we reduced the time spent for data annotation and introduced a sparse labeling technique, by which only a limited amount of data was labeled for training the model. We showed that U-Net with a combination of attention gates and residual links yielded the highest precision and accuracy compared to other tested architectures. This demonstrates that our approach, even with sparse labeling, can effectively identify blood vessels and provide accurate segmentation within tumor tissue. These findings are promising for improving our understanding of tumor vasculature and potentially contributing to improved treatment strategies.

**Abstract:**

Semantic segmentation is an important imaging analysis method enabling the identification of tissue structures. Histological image segmentation is particularly challenging, having large structural information while providing only limited training data. Additionally, labeling these structures to generate training data is time consuming. Here, we demonstrate the feasibility of a semantic segmentation using U-Net with a novel sparse labeling technique. The basic U-Net architecture was extended by attention gates, residual and recurrent links, and dropout regularization. To overcome the high class imbalance, which is intrinsic to histological data, under- and oversampling and data augmentation were used. In an ablation study, various architectures were evaluated, and the best performing model was identified. This model contains attention gates, residual links, and a dropout regularization of 0.125. The segmented images show accurate delineations of the vascular structures (with a precision of 0.9088 and an AUC-ROC score of 0.9717), and the segmentation algorithm is robust to images containing staining variations and damaged tissue. These results demonstrate the feasibility of sparse labeling in combination with the modified U-Net architecture.

## 1. Introduction

Deep learning is the leading artificial intelligence (AI) method for a wide range of tasks, including medical imaging problems. It is the state of the art for several computer vision tasks and has been used for medical imaging tasks, like the classification of Alzheimer’s, lung cancer detection, retinal disease detection, detection of abnormalities in modalities, like MRI, CT, X-ray, or ultrasound, and semantic segmentation of medical images [1,2]. Deep learning techniques have been used in the analysis of medical images in computer-assisted imaging contexts and offer a variety of solutions and improvements in the analysis of these images supporting radiologists and other specialists at diagnosis [3,4,5,6].

Semantic segmentation of vascular structures in histological images plays a pivotal role in various biomedical research fields and holds significant importance in advancing the understanding of complex biological systems. The intricate network of blood vessels is fundamental for tissue development and maintenance, as it facilitates the perfusion of nutrients and oxygen to cells. Accurately delineating and analyzing vascular structures are of paramount importance for the design and development of 3D in vitro systems that mimic the physiological conditions of human tissues [7,8,9,10].

In the field of image processing, the analysis of stained tissues to extract the information on vascular structures presents a formidable challenge due to the vast amount of data generated by high-resolution imaging techniques [11]. Additionally, vascular structures have to be segmented within in an ambiguous and heterogeneous tissue environment. Manual segmentation of vascular structures is labor-intensive, time-consuming, and prone to inter-observer variability [12].

**Figure 1:**
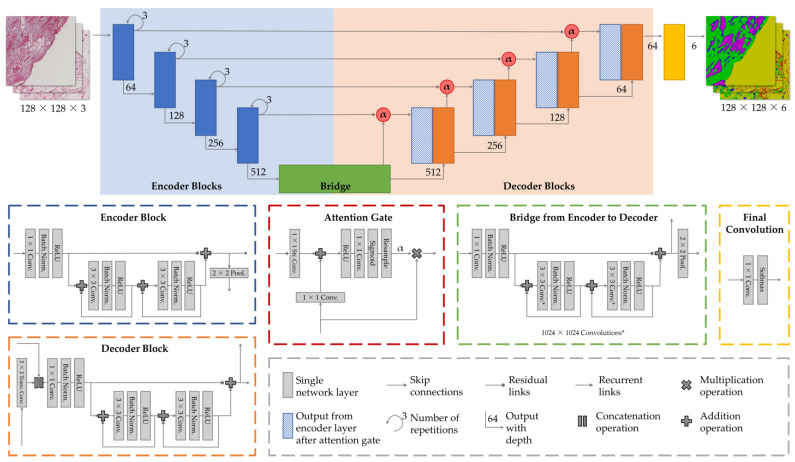
The network architecture. The network is based on the U-Net architecture, i.e., a four-level encoder/decoder convolutional network extended by attention gates, residual links, and recurrent blocks. The network segments six classes in histological tumor images. Arbitrary images can be handled by cutting them in patches with sizes of 128 px × 128 px × 3 (RBG color-coded), and after processing, stitching them back together to the original image. Details on the individual network architecture elements are given in the lower boxes. Their individual functionality is depicted within the main text (Section 2.3).

The need for automated and reliable segmentation algorithms is paramount to unlock the full potential of these large-scale datasets. Semantic segmentation techniques, particularly employing deep learning approaches, like U-Net [13], have demonstrated their capability in efficiently and accurately segmenting vascular structures from stained tissue images [14]. Such approaches enable handling “big data”, extracting valuable information from complex tissue samples, and advancing the understanding of disease processes [9], but there are several challenges that must be addressed, like limited data availability, overfitting of the network, or a high class imbalance in the training data. To overcome these challenges, this work uses a deep learning approach based on U-Net with sparsely labeled training data (see architecture in Figure 1).

Numerous applications can be identified, for which sophisticated semantic segmentation of vascular structures becomes vital. Notably, the development of 3D in vitro systems heavily relies on a comprehensive understanding of vasculature. These systems aim to mimic the intricate vascular architecture necessary for sustained cellular growth and function. Semantic segmentation of histological images enables the precise mapping of vascular networks within engineered tissues [15], aiding in the design and implementation of artificial perfusion to provide nutrients and oxygen to cells [16], thereby promoting their growth and viability. By replicating the intricate architecture of blood vessels, these systems can facilitate the perfusion of nutrients and efficient delivery of oxygen, closely resembling the conditions found in natural tissues. Another application is drug testing and development. Correctly delineating vascular structures in histology images aids in the development of 3D in vitro systems for drug testing [17,18]. By incorporating vascular networks into these systems, interactions of the therapeutic agents with blood vessels, drug efficacy, and potential side effects can be assessed [19]. This information is valuable for predicting drug behavior in the human body [20] and improving the accuracy of preclinical testing, ultimately leading to the development of safer and more effective treatments.

Image segmentation in general is an important task in computer vision, with applications in a variety of fields, including medical imaging and its analysis and processing, e.g., in computer-guided therapy and diagnostics, including:

1. Organ Segmentation: Segmentation of various organs in medical images, such as liver, kidney, pancreas, gall bladder, and lung [21,22,23]. This provides important support for diagnosing, surgical planning, and disease monitoring. However, these methods are based on CT or MRI data and are focused on localization of a target structure not on a precise segmentation [23].
2. Cell Segmentation: Segmentation of various cell types in microscopy images [24], such as detection of metastases in histopathology images [25], segmentation of cells’ nuclei [26,27], and segmentation of blood cells in microscopic images [28,29]. These methods are focused on localization rather than on an accurate segmentation and are applied to very homogeneous images.
3. Tumor Segmentation: Tumor segmentation in different types of medical images, such as ultrasound [30], computed tomography (CT) [31,32,33,34,35], and magnetic resonance imaging (MRI) scans [36,37]. Accurate segmentation of tumors is crucial for diagnosing, treatment planning, and monitoring of cancer patients. Conventionally, the image types for tumor segmentation are gained from ultrasound, CT, and MRI, showing characteristics that cannot directly be transferred to histological images.
4. Vessel Segmentation: Segmentation of blood vessels in various medical images, such as magnetic resonance angiography (MRA) scans [38], retinal images [39,40,41], and brain scans [42], which is important for the diagnosis and treatment of various diseases, such as diabetic retinopathy and stroke. Vessel segmentation is applied to various image modalities, yet the vessels are mostly homogeneous in structure. Typically, segmented images express two classes. Thus, for histological images, a scalable multiclass model that can be extended to classes specific to the desired application must be built.

Convolutional neural networks (CNNs) have shown remarkable success in image segmentation tasks, with the U-Net architecture being one of the most popular and effective CNN models for this task [14]. The U-Net architecture by Ronneberger et al. [13] is a U-shaped network that consists of an encoder and a decoder network connected by skip connections. The encoder network is a traditional CNN that consists of multiple convolutional and pooling layers. The decoder network is also a CNN, using transposed convolutions (also known as deconvolutions) to upsample the feature maps produced by the encoder network. One of the key features of the U-Net architecture is the use of skip connections between the encoder and decoder networks. These connections allow the decoder to access information from earlier layers of the encoder, which helps to preserve spatial information and leads to better segmentation results.

The focus of this work lies on the semantic segmentation with U-Net-based neural networks of blood vessels from histological human pancreatic tumor images. Pancreatic ductal adenocarcinoma is a type of cancer with limited treatment options due to its chemotherapy resistance [43]. Thus, new therapeutic options are required. Based on a dataset labeled with a novel sparse labelling technique, a set of 169 histological tumor images is fully segmented into six classes, two of which identify the vessels of the underlying tumor. Such data will provide valuable insights into tumor vascularization and angiogenesis and, furthermore, allow for in vitro and in silico analysis of tumor development and maintenance. U-Net has shown great promise for analyzing biomedical data [14]. Thus, to handle the described challenges, in addition to the original U-Net, this work incorporates attention mechanisms, dropout during the training to be more robust to the heterogenic data, a multiclass harmonic mean loss function, as well as data augmentation and under-/oversampling to resolve class imbalance. The semantic segmentation method is applied to histological tumor images. The modified U-Net architecture is discussed, and the results of the segmentation are evaluated using various metrics demonstrating precision of the segmentation results and thus its suitability for various applications.

All in all, this work introduces a deep neural network architecture that can segment histological images very precisely and thus enables various applications in the field of 3D in vitro systems in the generation of which detailed tissue structures are required. Additionally, a novel labeling technique that enables data annotation for segmentations of medical images within strongly reduced time and effort is introduced.

## 2. Materials and Methods

### 2.1. Sample Acquisition

With informed consent of the patient and permission of the local IRB (EK206/09), a specimen, a human pancreatic ductal adenocarcinoma, was resected and fixed in a 3.5% formaldehyde solution (Otto-Fischar GmbH, Saarbrücken, Germany), then embedded in paraffin wax, and sliced into 169 sections using a microtome (HM 340 E, Thermo Fisher Scientific Inc., Waltham, MA, USA) at a thickness of 2.5 µm per slice. These slices were then placed manually onto slides (Starforst, Engelbrecht Medizin- und Labortechnik, Edermünde, Germany) and dried overnight at 56 °C. Subsequently, the sections were stained with the “von Willebrandt factor” antibody (DakoCytomation, Glostrup, Denmark) using the ZytoChem Plus AP Polymer System (Mouse/Rabbit) (Zytomed Systems, Berlin, Germany) and Hematoxylin (Zytomed Systems, Berlin, Germany) and then covered with Vitro-Clud (R. Langenbrinck GmbH, Emmendingen, Germany). The slide images were digitalized using a microscope (Axio Imager.Z2, Carl Zeiss Microscopy Deutschland GmbH, Oberkochen, Germany) and cropped to a size of 13,020 × 10,422 px^2^, resulting in images with a pixel size of 0.5 µm.

### 2.2. Data Preparation

To reduce the network’s complexity, i.e., the number of trained parameters, the network’s input layer was limited to images with a size of 128 px × 128 px. To handle arbitrary image resolutions, images were cut into patches, which were processed independently and were re-aligned after processing and stitched back together. For the training, 7500 patches were randomly selected from the images and manually labeled.

All further preprocessing, the training steps were performed on these patches. For training, the patches were split into fractions of 60-20-20 for the training set, test set, and validation set, respectively. Data splitting is a fundamental step in machine learning model development, involving the division of data into training, validation, and test sets [44]. The training set is used to train the model by optimizing its parameters through iterative learning. The validation set serves as an independent subset for tuning hyperparameters and assessing the model performance during training. It aids in making informed decisions on model architecture and hyperparameter settings. Finally, the test set, which remains unseen during development, provides an unbiased evaluation of the model’s performance on real-world data. By using separate datasets, this data splitting strategy enables the prevention of overfitting, the assessment of generalization capabilities, and fair comparisons with other models or benchmarks.

To obtain reliable training data, there were many challenges that are especially prominent for deep learning applications in medical imaging. One major challenge was limited data availability, as only 169 slices from a single tumor were available, which makes it difficult to train deep learning models effectively. Another challenge was noisy and heterogeneous image data. In combination with limited data, this makes it challenging to train more universal models applicable to similar image data, as potentially many variations in image characteristics are not present in the training data. Class imbalance, i.e., the under-representation of important classes, is also a common issue in biomedical datasets [45] and was very prominent in the histological images introducing difficulties in-training models that can accurately classify all classes, e.g., pixels with very different characteristics belonging to the same class or vice versa. Lastly, a big challenge in deep learning was the need for large amounts of labeled data, which is time-consuming and expensive to obtain.

**Figure 2:**
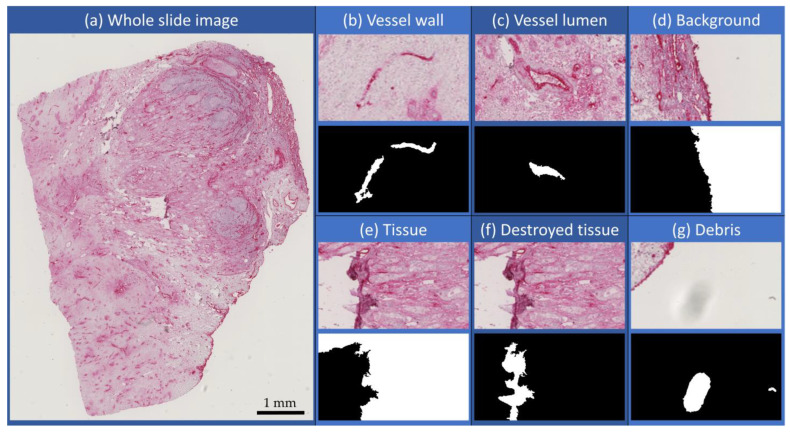
Input image and examples for each chosen class classification with an original excerpt on the top and the segmentation in black and white below. White shows the segmented regions of the respective classes, whereas black includes all other classes: (**a**) an exemplary whole-slide histological image with stained vessels; (**b**) two-vessel walls from elongated vessels; (**c**) vessel lumen with cells; (**d**) background; (**e**) intact tumor tissue; (**f**) corrupted/destroyed tissue; (**g**) debris on the slide (can also be occluded tissue). These examples show clearly that the segmented classes are very heterogeneous and not always distinguishable from one another without spatial localization.

**Figure 3:**
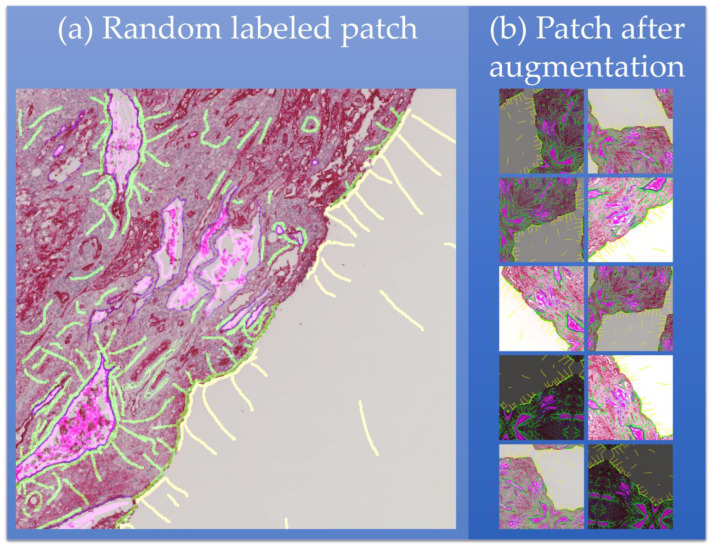
(**a**) Typical patch with sparse labeling and (**b**) patch after applying 10 different random augmentations with random contrast, brightness, zoom, rotation, and flip. When zooming or rotating, empty image parts are filled by mirroring, and the result is cropped to the original size of 128 px × 128 px. Each class is color-coded. Vessel walls: dark purple; vessel lumen: light purple; tissue: light green; background: yellow.

To cope with the abovementioned challenge of limited data, class imbalance, and labeling times, extensions to U-Net were incorporated, and a novel labeling technique was used. As vessels can take complex forms and are not homogeneous in their appearance, a distinction between vessel lumen and vessel wall was made. The remaining parts of the images were further subdivided into background, tissue, destroyed or corrupted tissue, and debris. Examples for each of those classes are shown in Figure 2b–g. Looking at the whole image slide in Figure 2a, the classes’ background and tissue are highly overrepresented, whereas the other classes, including the most relevant vessel lumen and vessel wall, are underrepresented leading to a high class imbalance. To counteract this imbalance, the overrepresented classes (background and tissue) were undersampled in labeling, and the classes’ vessel lumen and vessel wall were oversampled. Oversampling minority classes and undersampling majority classes are the best approaches to tackle class imbalance [46]. Both oversampling and undersampling were performed manually during labeling. Throughout testing, misclassified structures were identified, i.e., positive and negative hard mining [47,48], and added to the labeling to increase performance. This typically results in a patch as shown in Figure 3a. The labels in the exemplary patch show the sparse labeling used for the segmentation training. The classes’ vessel wall (dark purple) and vessel lumen (light purple) were extensively labeled, i.e., almost all pixels belonging to these classes were labeled for training. In contrary, the classes tissue (light green) and background (yellow) were highly undersampled. For this patch, this leads to a nearly equal label distribution for each class. During the manual labeling, there were intrinsic properties of the data that needed to be clearly identifiable by the network:

1. Vessel walls are fine but continuous structures: Walls are always labeled completely, indicating that there are no gaps, even with low image intensity.
2. Vessel lumens are separated from the tissue by a vessel wall: Vessel lumens are also labeled by drawing a line on the inside of the enclosing wall. Also, a line on the outside of the wall as tissue is labeled to indicate the separation from tissue and lumen by a wall. The remaining lumen is labeled by lines crossing the whole area of the lumen.
3. Remaining blood cells within the vessel lumen should be labeled as lumen: The labeled lines within the lumen go across the remaining cells to identify them as part of the lumen.
4. The tissue is a large continuous structure making up most part of the image: Tissue is labeled by long lines across the heterogeneous areas of tissue. These lines indicate its continuousness and penalize frequent changes in the segmentation.
5. The background is also large and continuous and located at the images’ edges: The background is also labeled with long lines across its area. A separating line at the tissue border indicates the transition from tissue to background.

These properties highlight the difficulties of segmenting vascular structures in a heterogeneous tissue environment and give indications on what aspects should be included during labeling.

As a final measure to handle class imbalance and lack of data, extensive data augmentation was applied to the training patches, which helped to increase the size of image datasets and improve model performance. U-Net’s architecture is particularly well-suited for data augmentation [13] because the skip connections allow for the incorporation of small image patches into the model without losing spatial resolution. This can help to address the challenge of limited data availability, as more training examples can be generated by applying transformations, such as rotations, flips, and zooms, to the original images [25,49]. This can improve the model’s applicability to similar data and its sensitivity to detect subtle features that may be missed by other architectures. Each patch was randomly augmented 10 times within the following parameter ranges:

1. Contrast: random increase or decrease in contrast by up to 90%;
2. Brightness: random increase or decrease in the brightness by up to 30%;
3. Zoom: random scaling between 50% and 200% of the original size, where x and y directions are scaled independently;
4. Rotation: random angle between −90° and 90°;
5. Flipping: no flipping, horizontal or vertical flipping.

Contrast and brightness are typical variations due to inhomogeneous staining of the tissue during preparation. Varying these aids the network in learning features that are invariant to contrast and brightness, such as vessel shapes or tissue borders. Zoom, rotation, and flipping additionally create more variation in the dataset counteracting the sparse data by introducing more feature variations. Figure 3b shows augmentations of an exemplary patch. Due to this manifold augmentation, the network becomes more robust when segmenting new images as more variations are seen during training than are in the labeled data. The 10 augmentations per patch lead to a total of 45000 training patches.

### 2.3. Network Architecture

The segmentation network is based on the original U-Net architecture introduced by Ronneberger et al. [13]. The U-Net architecture consists of an encoder network (Figure 1, blue), a bridge (Figure 1, green), and a decoder network (Figure 1, orange). The encoder network consists of a series of convolutional layers that progressively downsample the input image and extract high-level features. The output of the encoder network is a set of feature maps that represent the high-level features of the input image. The bridge connects the encoder network to the decoder network and typically consists of a single or double convolutional layer. The decoder network consists of a series of deconvolutional layers that upsample the feature maps to produce the final segmentation map. The decoder network is also characterized by its skip connections, which concatenate the feature maps from the encoder network with the upsampled feature maps from the decoder network. These skip connections allow the decoder network to recover spatial information from the encoder network and improve the segmentation performance. To improve the semantic segmentation of histological images, several extensions were considered that have proven effective when dealing with medical images.

#### 2.3.1. Attention Gates

Incorporating attention gates in the network provides the ability to selectively focus on regions of interest in the image [50,51]. The advantage of attention gates lies in their ability to assign different weights to different regions of the image based on their importance in the segmentation task [52] and thus aid in counteracting class imbalance. Attention gates also improve the model’s ability to handle complex images with high levels of noise, blur, or low contrast [14]. The application of attention gates in medical image segmentation has been shown to improve the segmentation accuracy by focusing the network’s attention on the most informative regions of the image [23,35,51,52]. Here, attention gates allow overweighting more complex classes, i.e., the fine-structured vessel wall and vessel lumen or the heterogeneous tissue, compared to a simple one, like the homogeneous background in the image (see Figure 1, dashed red box).

#### 2.3.2. Recurrent Links

**Figure 4:**
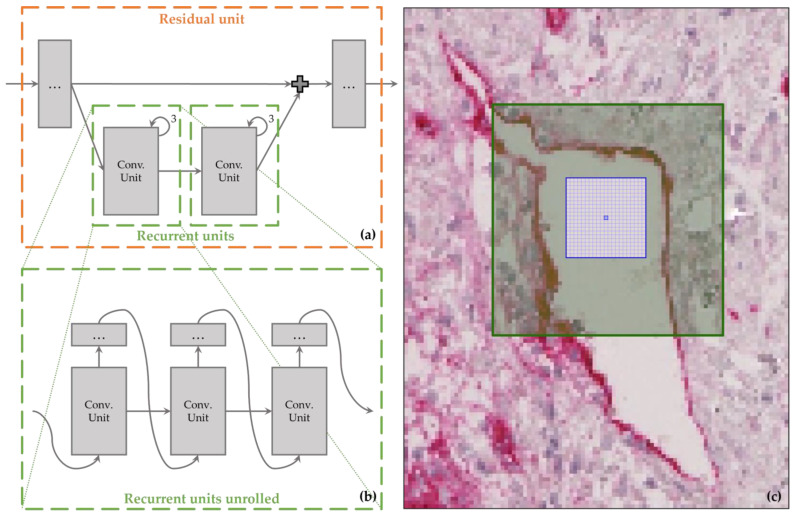
(**a**) The residual units bypass the two recurrent units in their encoder/decoder level (cf. Figure 1 for definition of symbols). (**b**) Unrolling the three-fold recurrent units visualizes the stacking of convolutions (conv.) and their effect on the receptive field. (**c**) Considering the blue pixel in the center, without recurrence, the networks receptive field corresponds to the blue grid of size 21 px × 21 px. Using recurrence increases the receptive field to the green area of size 61 px × 61 px, giving more spatial context to the convolutions.

Recurrent links allow the model to propagate information across the frames and capture the temporal dependencies between them [53]. The use of recurrent links in the network enables the incorporation of temporal information into the segmentation task. This is particularly useful in the case of histological images, where the segmentation over time of the vascular structures is an essential aspect of the segmentation task. Especially for the segmentation of histological images, recurrent links have proven useful and are widely incorporated in modern architectures [26,37,54,55,56]. As shown in Figure 4, each convolutional unit (3 × 3 convolution, batch normalization, activation) is extended to form a recurrent unit and can be repeated three times following the recurrent link.

The main purpose of recurrent links is capturing temporal dependencies; however, as a side effect of stacking 3 × 3 convolutions, the receptive field is also increased enabling the detection of larger structures. This becomes important when the network needs to distinguish a large vessel lumen from background. Using the formulas introduced by Araujo et al. [57], the receptive field for a pixel in the output layer can be computed as

(1) $$r_{0}=\sum_{l=0}^{L}\left((k_{l}-1)\prod_{i=1}^{l-1}s_{i}\right)+1$$

where $r_{0}$ stands for the receptive field in level 0 of the network, *L* for the total number of layers, $k_{l}$ for the kernel size used in layer *l*, and $s_{i}$ for the stride used in level *i*. Using (1) without recurrence, the receptive field of the blue pixel (Figure 4c) in the center is highlighted by the blue grid of size 21 px × 21 px. It can be observed that for the given pixel, the network has no spatial information to distinguish between background and vessel lumen. Introducing recurrence, the receptive field becomes larger, i.e., 61 px × 61 px, highlighted by the green square. The network now has far more spatial information and can more easily distinguish between the background and a lumen.

#### 2.3.3. Residual Links

The addition of residual links in the network preserves low-level (i.e., small-scale) features by allowing the network to bypass certain layers and propagate information directly from the input to the output [58]. So, similar to the skip connections where information from one encoder layer is passed directly to the corresponding layer in the decoder to preserve spatial information, the data are passed down/up to lower/higher layers (cf. Figure 1). This can be useful in medical image segmentation tasks, where small details, i.e., vascular structures, can be crucial in the future application. Additionally, residual links can prevent vanishing gradients, a common problem in deep learning networks with many layers, which may lead to a stop in a network’s learning after a certain number of layers [58]. Residual links have been shown to improve the segmentation performance in various medical image segmentation tasks [37,59,60,61], usually in combination with the other mechanisms (attention gates, recurrent links) incorporated here. In the implementation, each encoder/decoder block has one residual unit bypassing both its recurrent units (see Figure 4).

#### 2.3.4. Loss Function

A loss function is a crucial component in training a neural network as it quantifies the error between predicted and true values. In the case of segmenting histological images, a loss function that emphasizes accurate boundary prediction is necessary due to the complex and irregular shapes of the vascular structures. U-Net can be extended by a weighted loss function [62,63] to address class imbalance by using class weighting or by oversampling under-represented classes during training [46,64,65]. This can help to ensure that the model is able to accurately classify all classes, even those that are less frequent in the training data. Two of the most commonly used error metrics are precision and recall [66]. Precision (PREC, Equation (2)) describes the fraction of correctly identified pixels among all pixels predicted for that class, whereas recall (RECL, Equation (3)) is the fraction of correctly identified pixels among all pixels labeled for that class:

(2) $$PREC=\frac{TP+\sigma }{TP+FP+\sigma }$$

(3) $$RECL=\frac{TP+\sigma }{TP+FN+\sigma }$$

Here, *TP* stands for true positive, i.e., all correctly predicted pixels, *FP* (false positive) for all pixels from another class predicted to be in the considered class, and *FN* (false negative) for all pixels of the considered class predicted to be in another class. σ is a smoothing operator for numerical stability. Combining both metrics with a harmonic mean yields the typically used F1 score or Dice score [54]:

(4) $$DICE=\frac{2+\sigma }{\frac{1}{PREC}+\frac{1}{RECL}+\sigma }=\frac{2TP+\sigma }{2TP+FP+FN+\sigma }.$$

As this work proposes a multiclass segmentation network, the loss function evaluates the Dice score for each of the six classes separately, combines them by a harmonic mean and turns the score into a loss. A harmonic mean is very sensitive to variations in each of its values, so as a loss function, it penalizes unprecise segmentations and misclassifications and forces the network to minimize the classification error on all classes, which is needed here due to a high class imbalance. So, for each class c∈C where *C* is the set of the defined six classes, the total loss of the network can then be calculated using

(5) $$L=1-\frac{\left|C\right|}{\sum_{c\in C}\left(\frac{1}{{PREC}_{c}}+\frac{1}{{RECL}_{c}}+\frac{1}{DICE_{c}}\right)}$$

thus, combining all individual class scores and normalizing the loss to the range [0, 1].

In addition to the metrics precision, recall, and Dice, a fourth metric called specificity [66] (also called inverse recall) is measured. The specificity gives the true negative (*TN*) rate, i.e., the fraction of correctly identified pixels among all pixels labeled as not belonging to that class and is defined as

(6) $$SPEC=\frac{TN+\sigma }{TN+FP+\sigma }$$

The specificity is not part of the loss function but is used as an evaluation metric.

#### 2.3.5. Hyperparameters

Hyperparameters determine the behavior of the training algorithm and directly affect the performance of the model. Performing a grid search on hyperparameters is essential, regarding the defined loss function, to identify the optimal configuration of hyperparameters for a deep learning network.

In the proposed deep learning network, a grid search on five hyperparameters was performed: batch size, dropout, learning rate and the activation of attention gates, residual links, and recurrent links. For the batch size, 1, 2, 4, 8, 16, and 32 were tested. For the dropout, four values, i.e., 0, 0.125, 0.25, and 0.375, were tested. For the learning rate, 1 × 10^−2^, 1 × 10^−3^, and 1 × 10^−4^ were tested. Additionally, the activation of attention gates, residual links, and recurrent links were tested as binary options. All combinations of these hyperparameters were assessed using a training and validation set, and the optimal configuration of hyperparameters was selected based on the performance metrics on the validation set. The optimal hyperparameters according to multiple runs of this grid search were identified as:

- Batch size: 16 (for all architectures);
- Dropout: 0.25 (for Recurrent U-Net) and 0.125 (for all other architectures);
- Learning rate: 1 × 10^−4^ (for all architectures).

The implementation uses a dynamic learning rate approach for the deep learning model. Specifically, the initial learning rate is set to 1 × 10^−4^, according to the grid search, but instead of using a fixed learning rate throughout the training process, it is reduced by a factor of 0.1 whenever the loss of the network plateaued for 10 epochs. This approach allowed fine-tuning the learning rate as the model learned more about the data and prevented the model from getting stuck in sub-optimal local minima. Additionally, early stopping was employed to further prevent overfitting of the model. After four consecutive plateaus in the loss, the training process was stopped, with a maximum of 300 epochs.

The use of dropout regularization in the network also prevents overfitting by randomly dropping out a fraction of the layer outputs during training, thus, forcing the network to learn more robust features. Dropout regularization prevents the network from memorizing the training data and improves the generalization performance of the model [67,68]. After each encoder and decoder block, the respective dropout is applied.

### 2.4. Used Software and Hardware

For the development of the network, the open-source TensorFlow library and Keras library together with TensorBoard for evaluation and visualization of the network’s performance were used [69,70]. The evaluation graphs were created with matplotlib [71]. Data labeling was performed manually with the Medical Image Labeler from MATLAB’s Medical Imaging Toolbox™ (The MathWorks, Inc., Natick, MA, USA).

For training, all computations were performed with computing resources granted by RWTH Aachen University. The RWTH Aachen University High Performance Cluster (RWTH Aachen University, Aachen, NRW, Germany) provides access to computational nodes with access to 2 GPU-cards of type V100-SXM2.

## 3. Results

In this section, results of a quantitative analysis of an ablation study are presented, giving an overview of the network’s performance. The common coefficients Dice, precision, recall/sensitivity, and specificity are used for evaluation. In the ablation study, each of the added architectural mechanisms, namely attention gates, residual links, and recurrent links, are tested individually and in combination. Additionally, the number of training parameters are listed for further comparison among the different models. Furthermore, to assess the segmentation separately for each class, AUC ROC evaluations, elevated for a multiclass approach, are presented. In a qualitative analysis, the segmentations are compared visually, indicating strengths and deficits of each model.

### 3.1. Quantitative Ablation Study

From the perspective of a quantitative analysis, an ablation study for six different architectural variants was performed and evaluated. From the results shown in Table 1, it becomes evident that the sparse labeling in combination with different U-Net architectures yields very accurate segmentation results on histological data. The performance results of each architectural variant are summarized in Table 1. Additionally, in Supplement Figure S1, a confusion matrix for each architecture is given. The baseline model, a basic U-Net, already achieved high scores with a precision of 0.9032, a recall of 0.8601, a specificity of 0.9877, and a Dice coefficient of 0.8432. Introducing attention gates to the U-Net architecture led to slight improvements in precision (0.9053) and recall (0.8621), while maintaining a high specificity of 0.9864. The Dice coefficient increased to 0.8524, indicating an enhanced segmentation performance. Incorporating residual links into the U-Net architecture impacts the precision slightly, decreasing it to 0.8961, but the recall remained reasonable at 0.8435. The specificity remained high at 0.9879. However, the Dice coefficient showed a moderate decrease to 0.8197, suggesting a slight reduction in segmentation accuracy compared to the baseline. The addition of recurrent links in the U-Net architecture resulted in a lower precision of 0.8058 and recall of 0.8080. Despite this, the specificity remained high at 0.9782. However, the Dice coefficient noticeably decreased to 0.7436, indicating a notable decrease in segmentation accuracy compared to the basic U-Net.

Incorporating each of the presented architectural methods independently already suggests that the segmentation benefits from added attention gates and residual links. Adding recurrence, however, decreases the segmentation performance. The reason for this is overfitting. The more complex network can more easily learn the small dataset, as it has far more parameters than the other architectures: 35.6 million >> 31.1 million for the baseline model. Next to the number of parameters, the number of training epochs also indicates overfitting. Although the recurrence model is more complex, it takes over 100 epochs less for training, indicating an overfit to the training set. Lastly, the recurrent U-Net was the only architecture where the best dropout was identified as 0.25 (instead of 0.125), which also implies overfitting.

Combining attention gates with residual links yielded a higher precision of 0.9088, albeit with a slightly lower recall of 0.8383. The specificity remained consistently high at 0.9869. However, the Dice coefficient decreased to 0.8247, suggesting a moderate reduction in segmentation accuracy compared to the basic U-Net. Finally, incorporating attention gates, residual links, and recurrent links in the U-Net architecture led to the lowest precision (0.7974), recall (0.8117), and the Dice coefficient (0.7432). However, the specificity remained high at 0.9787. As before, the network performs well, yet with the same argumentation as before, overfitting to the small dataset has to be assumed.

Based on the quantitative analysis, the U-Net model with attention gates demonstrated improved precision, recall, and the Dice coefficient compared to the basic U-Net (cf. Table 1). The addition of residual links had varying impacts on the performance, and the inclusion of recurrent links generally resulted in lower segmentation accuracy, which is attributed to overfitting. Notably, the combination of attention gates and residual links provided a trade-off between precision and the Dice coefficient. However, the model with attention gates, residual links, and recurrent links did not perform as well, showing lower precision and Dice coefficient.

**Figure 5:**
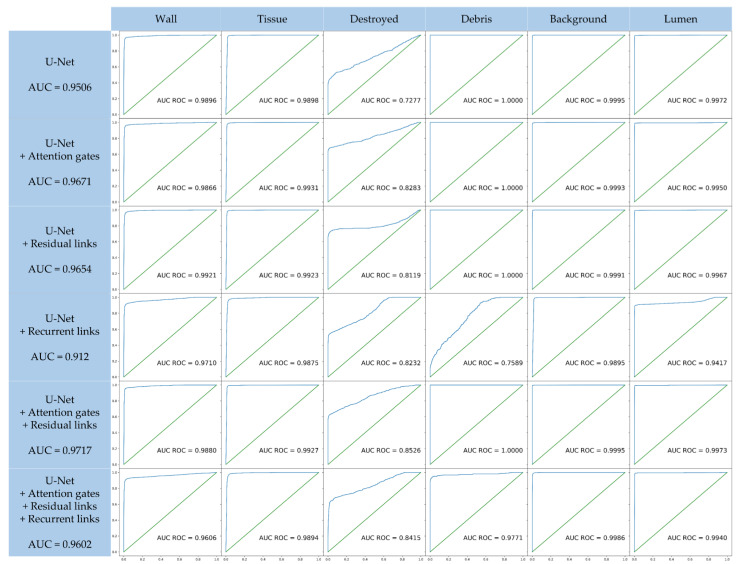
Comparison of the ROC curves with their AUC scores for each architecture and each individual class. The ROC curves for each class were computed using the one-vs-rest method, which indicates how good the respective network can distinguish the individual class from the other classes. Each diagram shows the AUC-ROC score for the specific combination of architecture and selected class. The AUC scores beneath the architecture configurations in the first column are the total AUC-ROC scores for the multiclass evaluation.

In addition to these global metrics that assess the segmentation as one, Figure 5 shows the AUC ROC curves [72] for each class. Here, the computation for ROC curves for binary classification estimation was elevated to fit the multiclass approach by the one-vs-rest method. The curves in Figure 5 clearly show that the segmentation algorithm performs well on all defined classes as the total AUC ROC values are all above 0.912. Apart from the class for destroyed tissue, all classes are segmented reliably as indicated by their AUC ROC, which was very close to 1. This was consistent for all models, indicating that there were too little training data for the class of destroyed tissue.

This class is very heterogeneous (cf. Figure 2f) and thus needs more training data to cover all class characteristics. Still, the overall performance of all models is very good. The basic U-Net sets a high baseline with a total AUC score of 0.9506. With added attention gates, the model improved its performance (AUC score of 0.9671), especially on the class of destroyed tissue. Residual links also increased the performance to an AUC score of 0.9654. As seen before, the added recurrent links decreased the performance of the model, reaching the lowest AUC score of 0.912. Although the model achieves good performance on the class of destroyed tissue, the performance on the classes of vessel walls, vessel insides, and debris significantly decreased compared to the baseline model. Being the only model that performs poorly on the class of debris again implies overfitting as seen before. The class of debris is the smallest labeled class and thus can be more easily memorized by the more complex model. The combination of attention gates and residual links led to the highest AUC score of all models of 0.9717. The model achieved the best performance on almost all individual classes. Only the classes of vessel wall and tissue are surpassed by the baseline model with 0.0016 and the attention model with 0.0004, respectively. The model with attention gate and residual and recurrent links also showed an increased performance (AUC score of 0.9602) compared to the baseline model. It showed a slightly reduced performance on the class of vessel walls yet achieves the second-to-best score on the class of destroyed tissue. Also, the decrease in the class of debris that was expressed in the model with only recurrent links is compensated through the attention gates and residual links, thus showing great promise for a better performance on a larger dataset with increased yet sparse labeling.

### 3.2. Qualitative Segmentation Analysis

**Figure 6:**
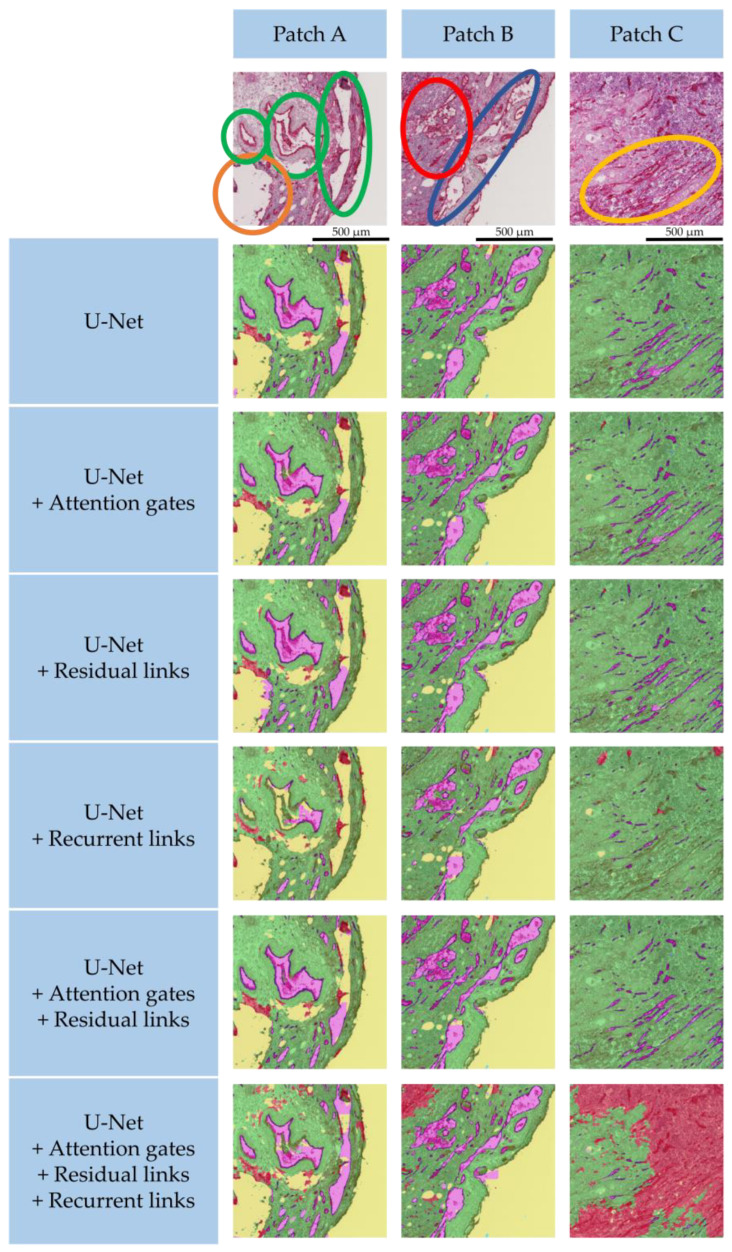
Qualitative segmentation results of all models shown exemplarily on three different patches. The patches on top are taken from original images. The results below show overlays of the respective patch with its segmentation result. Vessel lumens are colored purple, vessel walls are dark blue, tissue is green, destroyed tissue is red, and the background is yellow. Beneath each architecture, the AUC-ROC scores from Figure 5 are included for comparison.

The qualitative results are shown in Figure 6. Three patches are exemplarily shown to highlight the strengths and deficits of each model. Patch A shows three large and prominent vessels (marked with green circles), color variations from very bright on the left side to very dark on the right side, as well as some destroyed tissue in the bottom left area (marked with an orange circle). Patch B has also strong color variation from dark on the left to bright on the right. There are a lot of complex shaped vessels in the patch’s center (marked with blue circle) that are very close to one another and contain a lot of structures (e.g., blood cells) showing a very heterogeneous lumen (marked with red circle). Patch C is very dark with little contrast. There are many elongated small vessels present with no lumen (marked with yellow circle).

For patch A, the U-Net baseline model clearly segments most vessels with their lumen in purple and their wall in dark blue. Also, the destroyed tissue in the bottom left area and in the center was correctly identified (marked red), and the tissue (marked green) covers all remaining parts of the tumor slice and has a sharp border to the background (marked yellow). However, the elongated vessel on the right is only partially segmented and was falsely identified as background. For patch B, the complex shapes and the heterogeneous lumens were correctly identified. Also, patch C shows great results, as the elongated structures are finely separated from one another.

With added attention gates, the visual results look very similar to the reference model. Vessel walls and lumens were well segmented in all three patches. The model identified more of the destroyed tissue in patch A, which is consistent with the models increased AUC-ROC score in Figure 5 for this class, as seen before. In the center of patch A, the vessel contains some blood cells that were misclassified as tissue.

The same holds for the added residual links. In patch A, the model falsely identified some vessel lumens in the destroyed tissue region but correctly segmented the vessel lumens of the center vessel.

As the metrics in Table 1 reflect, the segmentation of the U-Net with added recurrence performs less accurately than the reference model. The large vessels in patch A were missed almost completely. Also, patch B and C are missing vessels and have only partially segmented lumens. The model also falsely identified a lot more destroyed tissue, especially in the dark regions in patch C.

The combination of attention gates and residual links shows very accurate segmentations. In all patches, the complex-shaped or small vessels are identified correctly. In patch A’s center vessel, the heterogeneous blood cells, apart from very few pixel errors, are completely segmented as part of the lumen. There is also more identified, destroyed tissue than in the reference model. These good results are again consistent with the metrics in Table 1 and the AUC-ROC scores in Figure 5.

Lastly, the model with added attention gates and residual and recurrent links shows good segmentations of vessels in patches A and B. In the center vessel of patch A, the heterogeneous region was partially misclassified as tissue; however, this model is the only one that almost completely identified the homogeneous elongated vessel on the right of patch A. Yet, there is an overexpression of destroyed tissue. This model tends to classify dark regions as destroyed tissue, which is very prominent in patch C. As before, this is a clear sign of overfitting, as most destroyed regions in the dataset are dark and heterogeneous. Bigger training data for such regions will be necessary to avoid the overfitting for this complex model.

### 3.3. Selection of the Best Architecture

**Figure 7:**
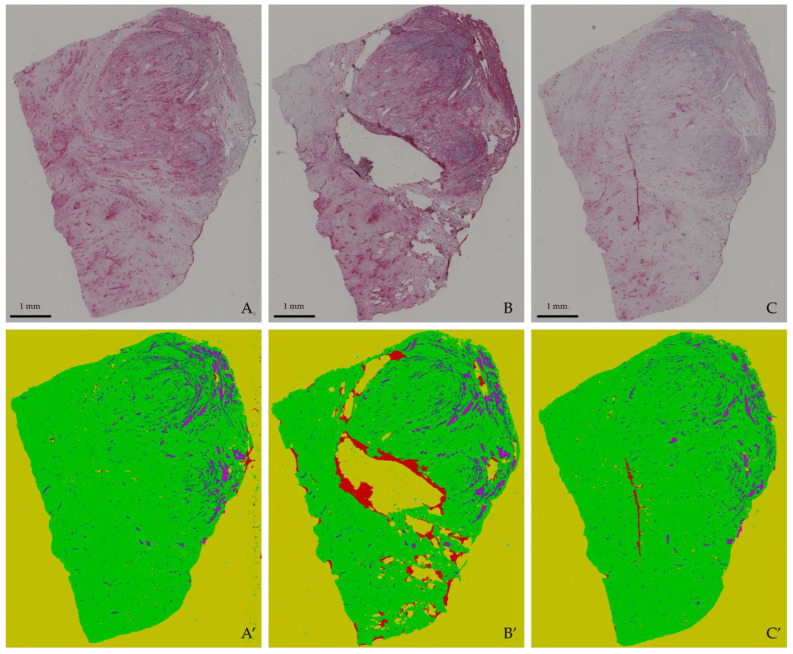
Three full slices segmented using U-Net with attention gates and residual links. The slices give examples of slices with high contrast (**A**), slices with intensity variations and a lot of destroyed tissue (**B**), and very bright slices with very little contrast (**C**). The model performs well on all of these difficulties. The segmentations are shown below and are denoted (**A’**), (**B’**), and (**C’**).

The quantitative analysis showed that each of the presented architectures had their individual benefits and drawbacks. The attention gates achieved the highest scores for recall and Dice, whereas residual links achieved the highest sensitivity. The combination of both mechanisms provided a great trade-off between these scores and achieved the best precision. Although the recurrence increased the receptive field and thus yielded a good performance on large homogeneous structures as seen in Figure 6, its performance metrics were the lowest among all tested architectures, which is attributed to overfitting the small dataset. Yet, the analysis of the AUC-ROC scores showed promising qualities for including recurrence in future models, as its ability to distinguish between the different classes among the other models. With extended computational resources and further labeling, further investigations using recurrent links in semantic segmentation models will enable an even better segmentation of heterogeneous structures. The metrics in Table 1, the AUC-ROC scores in Figure 5 and the visual inspection in Figure 6 demonstrate that the model combining attention gates and residual links yields the most promising results for a semantic segmentation of histological images. Figure 7 shows three full slices from the dataset with their respective segmentation. Clearly defined structures with sharp edges and very few misclassifications allow the best applicability of these segmentation results for subsequent quantitative analysis, clinical decision-making, and advanced image-guided interventions.

## 4. Discussion and Future Work

With this study, we presented a model that handles histological images with high contrast (Figure 7A), intensity variations, and destroyed tissue (Figure 7B), as well as very bright slices with little contrast (Figure 7C). The model was designed for segmenting histological images, yet the presented methods are versatile and can be applied to all sorts of applications in medical image segmentation. Depending on the desired application, more classes (e.g., for glands, cell nuclei, etc.) can be designed and given as new training input. To the best of our knowledge, there are no semantic segmentation algorithms that can achieve this level of accuracy and precision when delineating vascular structures in histological images. Having a precise segmentation across consecutive slices makes it possible to trace the vessels’ paths through the tissue and potentially reconstruct them in a 3D model. To track the vessels’ paths through the tissue, it is important that the whole slide images are registered. Several registration techniques designed to re-align medical images are available [73,74,75,76,77,78,79,80,81,82,83], many of which are based on SIFT [84] or SURF [85] feature point extraction, which can also be applied to histological images with stained vessels [86]. To overcome the challenge of feature extraction in highly repetitive patterns, normalized cross-correlation was used [87,88], and to improve the similarity measure, the Gaussian Laplacian second-order differential operator was additionally applied [89].

To implement the described deep learning approach, data scarcity was a major challenge in this work. The used dataset consisted of 169 histological slices that had no annotations. Fully annotating them manually is very time-consuming and would require medical experts. Therefore, a novel sparse labeling technique (cf. Section 2.2) was employed in this work. This new labeling technique enabled us to annotate a reasonably large dataset within a feasible timeframe to be able to train a deep neural network. Approaches, like unsupervised domain adaptation (UDA) [90], address the challenge of data annotation. UDA leverages knowledge from a labeled source domain, performing a so-called domain shift, to improve model performance on an unlabeled or sparsely labeled target domain. [91] In the context of medical imaging, UDA has gained increasing attention to overcome the limitations of collecting large, labeled datasets across different medical imaging modalities [90,91]. However, it is important to note that UDA may not be directly applicable to histological datasets in certain cases. Histological images present unique challenges due to their high complexity, fine-grained structures, and significant staining variations. The domain shift between different histological datasets can be highly complex and difficult to capture solely through unsupervised adaptation techniques. There are approaches to handle these challenges of complex domains via orthogonal decomposition of domain specific features [92] or disentanglement of domain invariant and domain specific modalities [93]. There are UDA approaches dealing with histological images, i.e., for general whole-slide images [94] or specific to the classification of breast cancer images [95]. Further advancements bridging domains of different imaging modalities are made [96] by, e.g., style adaptation [97] or selective entropy constraints [98] where pixels are first categorized in reliable and unreliable before the domain shift. However, all these approaches rely on a well-labeled source domain. This work targets applications that include, e.g., 3D reconstruction of in vitro models, for which a very precise pixel-wise segmentation is needed. To the best of our knowledge, there are no publicly available datasets with such detailed annotations available. In such cases, alternative approaches are more appropriate for addressing the domain shift and improving the performance of models on histological datasets. Therefore, we introduced a new labeling technique that was specifically tailored to address the identified challenges of data scarcity, class imbalance, and the data’s heterogeneous structure.

With the labeled dataset, the network presented in this work was able to segment the images into six classes with high precision (cf. Table 1). The two most interesting classes regard the vascular structures of the used specimen. There are many applications, in which delineating vessels with various imaging modalities plays an important role, e.g., for early detection of severe diseases, like diabetes, hypertensive retinopathy, or retinal vessel segmentation [39,40,41]. Retinal images, however, consist of long and fine-grained homogeneous structures with significant lower complexity than histological tissue images. Other applications are cerebrovascular diseases, for which the detection of deformations and abnormalities in brain blood vessels with digital subtraction angiography (DSA) images is of paramount importance [42]. Similar to retinal images, DSA images are homogeneous and contain long vessel structures. More complex or heterogeneous images are encountered in X-ray angiography (XRA) segmentation. The segmentation of main coronary arteries is important for assessing coronary diseases, such as stenosis [99]. So far, a progressive perception learning framework was proposed that segments coronary arteries using boundary perception [100]. All these applications are based on image modalities containing significantly less complex vessel information. DSA, XRA, MRI, or CT have too low resolutions to capture small arteries of down to 10 nm [101,102]. This makes the model presented in this work unique in the reconstruction of 3D in vitro networks with a resolution and structural information.

However, the model accuracy may benefit from improvements. Especially concerning the areas of destroyed tissue or low contrast regions, the distinction between vessels and tissue remains challenging. By expanding the training dataset and incorporating more patches with these challenging areas, we are confident to further increase the model performance. Having more data will also allow to further incorporate recurrent links into the architecture, which was not feasible due to the identified overfitting.

Concerning the implementation of the model and its clinical use, code verification and security is required. Here, the application of formal methods and smart contracts offers a novel approach to enhance the verification and correctness of deep learning code. In the context of this work, the following ideas could be applied. By leveraging the transparency and immutability of blockchain technology, smart contracts could provide several advantages in ensuring code integrity [103,104,105]. Different formal methods, such as model checking or runtime verification, could be applied. With model checking [106,107,108], storing the deep learning code and model parameters on the blockchain could enable the verification of code execution by comparing the recorded hash or identifiers with the executed results. This would allow for easy detection of discrepancies or deviations, ensuring the correctness of the code execution. This could also facilitate the validation of model training. Recording essential training details on the blockchain, such as datasets, hyperparameters, and model architecture, could increase transparency and external verification. This would guard against manipulation or data leakage, enhancing the reliability and correctness of the model training process. By runtime verification [106,109], the continuous monitoring of model performance is made possible. By periodically receiving model predictions or metrics stored on the blockchain, comparisons with predefined benchmarks or ground truth data can be made. This could enable the early detection of issues, like model degradation or adversarial attacks, ensuring the ongoing correctness of the deep learning model’s performance. Implementing such formal methods to ensure code verification, training validation, performance monitoring, and model governance will be an important part of our ongoing development for a future clinical setting. These proposed methods are ideas on how smart contracts could be used to verify a deep learning model within a sensitive medical context, as this is an emerging research field and there is no standard procedure for deep learning verification yet.

## 5. Conclusions

In conclusion, the deep learning network for semantic segmentation of vascular structures in histological images incorporates several techniques to improve the segmentation performance. The addition of attention gates, residual links, and recurrent links allows the network to selectively focus on regions of interest, preserve low-level features, and model the segmentation over time of the vascular structures. The use of data augmentation, under-/oversampling, and dropout regularization addresses class imbalance in the data and prevents overfitting in the network, respectively. The combination of these techniques demonstrates the feasibility of sparse labeling and thus introduces a novel labeling technique applicable for biomedical data, where a precise labeling in itself is an elaborate task and results in a deep learning network that exceeds state-of-the-art segmentation algorithms on the task of semantic segmentation of histological images.

## Figures and Tables

**Table 1:** Performance comparison for each variant investigated in the ablation study. Each variant was trained three times, and the values are reported as average and standard deviation. Bold numbers indicate the best performance for the respective metric. Precision, recall, specificity, and Dice range from 0 to 1, with 1 being a perfect segmentation with no errors. See Supplementary Materials for the confusion matrices leading to these evaluations.

Method/Metric	Precision (Equation (2))	Recall/ Sensitivity (Equation (3))	Specificity (Equation (6))	Dice (Equation (4))	Trained Parameters	Trained Epochs	Dropout Regularization
Basic U-Net	0.9032 (± 0.0144)	0.8601 (± 0.068)	0.9877 (± 0.007)	0.8432 (± 0.0657)	31,055,622	212	0.125
U-Net + Attention gates	0.9053 (± 0.0069)	**0.8621 (± 0.0041)**	0.9864 (± 0.0008)	**0.8524 (± 0.0022)**	31,778,762	245	0.125
U-Net + Residual links	0.8961 (± 0.0048)	0.8435 (± 0.0129)	**0.9879 (± 0.001)**	0.8197 (± 0.0148)	32,463,174	191	0.125
U-Net + Recurrent links	0.8058 (± 0.0481)	0.8080 (± 0.0113)	0.9782 (± 0.0038)	0.7436 (± 0.0415)	35,631,750	108	0.25
U-Net + Attention gates + Residual links	**0.9088 (± 0.0061)**	0.8383 (± 0.019)	0.9869 (± 0.0005)	0.8247 (± 0.0235)	33,186,314	217	0.125
U-Net + Attention gates, Residual links, Recurrent links	0.7974 (± 0.0074)	0.8117 (± 0.024)	0.9787 (± 0.0005)	0.7432 (± 0.0187)	37,762,442	86	0.125

## Data Availability

Data are available in a pseudonymized manner upon request due to ethical restrictions (patient confidentially). Therefore, the data are not available publicly.

## Supplementary Materials

The following supporting information can be downloaded at: https://www.mdpi.com/article/10.3390/cancers15153773/s1, Figure S1: The confusion matrices for each of the evaluated network architectures.
