# Supplementary material for: Vessel Delineation Using U-Net: A Sparse Labeled Deep Learning Approach for Semantic Segmentation of Histological Images
Source: Cancers (Basel). 2023 Jul 25;15(15):3773. doi: 10.3390/cancers15153773 (PMC10417575; doi:10.3390/cancers15153773)
Supplement: Supplementary file 1 [file cancers-15-03773-s001.zip › cancers-2507323-supplementary.pdf]

Supplementary data

Vessel delineation using U-Net: A sparse labeled deep learning approach for semantic segmentation of histological images

Lukas Glänzer [1], Husam E. Masalkhi[1], Anjali A. Roeth[2,3], Thomas Schmitz-Rode[1] and Ioana Slabu[1,] *

[1] Institute of Applied Medical Engineering, Helmholtz Institute, Medical Faculty, RWTH Aachen University, Pauwelsstraße 20, 52074 Aachen, Germany
[2] Department of Visceral and Transplantation Surgery, University Hospital RWTH Aachen, Pauwelsstrasse 30, 52074 Aachen, Germany
[3] Department of Surgery, Maastricht University, P. Debyelaan 25, 6229 HX Maastricht, The Netherlands
* Correspondence: slabu@ame.rwth-aachen.de; Tel.: +49 241 80 89102 (I.S.)

S1. Composition of the evaluation metrics

The results presented in Table 1 show evaluation metrics precision, recall, specificity and Dice alongside the number of training parameters, number of trained epochs and the dropout regularization applied for the specific model. The number of trained parameters and number of epochs are properties that were tracked by the implementation and the dropout value was determined within the ablation study presented in section 3. The evaluation metrics, however, were computed by comparing the computed segmentation to the initially given ground truth, i.e. the labeling of the classes. Figure S1 presents the confusion matrices for each of the considered architectures. The metrics in Table 1 were then computed based on these matrices.

U-Net

Labeled classes \ Predicted classes	Vessel wall	Tissue	Destroyed tissue	Debris	Background	Vessel lumen
Vessel wall	2592	139	0	0	0	161
Tissue	347	48299	19	0	245	59
Destroyed tissue	0	420	219	0	0	0
Debris	0	0	0	250	0	0
Background	0	110	1	1	16732	39
Vessel lumen	73	0	0	0	0	6249

U-Net + Attention gates

Labeled classes \ Predicted classes	Vessel wall	Tissue	Destroyed tissue	Debris	Background	Vessel lumen
Vessel wall	2556	148	0	0	0	188
Tissue	384	48238	7	0	238	102
Destroyed tissue	2	280	357	0	0	0
Debris	0	0	0	250	0	0
Background	0	132	0	0	16673	39
Vessel lumen	77	0	0	0	0	6245

U-Net + Residual links

Labeled classes \ Predicted classes	Vessel wall	Tissue	Destroyed tissue	Debris	Background	Vessel lumen
Vessel wall	2575	153	0	0	0	164
Tissue	387	48247	0	0	311	24
Destroyed tissue	0	303	336	0	0	0
Debris	0	0	0	250	0	0
Background	0	90	0	13	16741	0
Vessel lumen	64	1	0	0	29	6228

U-Net + Recurrent links

Labeled classes \ Predicted classes	Vessel wall	Tissue	Destroyed tissue	Debris	Background	Vessel lumen
Vessel wall	2470	279	0	0	1	142
Tissue	363	47299	167	0	1137	3
Destroyed tissue	0	269	307	0	63	0
Debris	0	103	0	8	139	0
Background	0	92	0	0	16752	0
Vessel lumen	122	68	0	0	660	5472

U-Net + Attention gates + Residual links

Labeled classes \ Predicted classes	Vessel wall	Tissue	Destroyed tissue	Debris	Background	Vessel lumen
Vessel wall	2597	129	0	0	0	166
Tissue	457	48346	0	0	113	53
Destroyed tissue	0	324	306	0	9	0
Debris	0	0	0	249	1	0
Background	0	97	0	1	16746	0
Vessel lumen	71	0	0	0	0	6251

U-Net + Attention gates + Residual links + Recurrent links

Labeled classes \ Predicted classes	Vessel wall	Tissue	Destroyed tissue	Debris	Background	Vessel lumen
Vessel wall	2286	319	4	0	0	283
Tissue	238	47544	437	0	523	227
Destroyed tissue	0	336	303	0	0	0
Debris	0	2	58	178	12	0
Background	0	170	1	15	16620	38
Vessel lumen	59	10	2	0	0	6251

Figure S1. The confusion matrices for each of the evaluated network architectures.
